# Surgery for primary ventral hernias and risk of postoperative pain, nausea: a population-based register study

**DOI:** 10.1007/s10029-025-03256-4

**Published:** 2025-01-15

**Authors:** Fathalla Ali, Göran Wallin, Rebecka Rubenson Wahlin, Agneta Montgomery, Peder Rogmark, Gabriel Sandblom

**Affiliations:** 1 Faculty of Medicine and Health, Department of Surgery, Örebro University Hospital, Örebro, Sweden; 2 Division of Anesthesiology and Intensive Care, Department of Clinical Science, Technology and Intervention, Karolinska Institutet, Huddinge, Sweden; 3 Department of Surgery, Skane University Hospital, Malmö, Sweden; 4 Department of Clinical Sciences, Malmö, Lund University, Lund, Sweden; 5 Department of Clinical Science and Education Södersjukhuset, Karolinska Institutet, Stockholm, Sweden; 6 Department of Surgery, Södersjukhuset, Stockholm, Sweden

**Keywords:** Primary ventral hernia, Umbilical hernia, General anesthesia, Inhalation anesthesia, Postoperative pain and nausea/vomiting

## Abstract

**Purpose:**

The aim of this study was to evaluate risk factors for postoperative pain and nausea after open repair for primary ventral hernias.

**Method:**

A population-based registry study was conducted based on data assembled from the Swedish national ventral hernia repair register between January 2016 and December 2021and cross-matched with the Swedish perioperative register.

**Results:**

Altogether 2064 open ventral hernia repairs were registered, including 816 (39.5%) performed on women. Of these, 91 (4.4%) were registered to suffer postoperative nausea or vomiting (PONV) and 403 (19.5%) postoperative pain (PP). In both univariable and multivariable logistic regression analyses, significant predictors of postoperative nausea and pain included male gender, which was associated with lower odds of both postoperative nausea (multivariable OR: 0.30, 95% CI: 0.18–0.49, *P* < 0.001) and postoperative pain (multivariable OR: 0.60, 95% CI: 0.44–0.83, *P* = 0.002). Additional predictors of postoperative nausea included emergency surgery (multivariable OR: 4.08, 95% CI: 1.10-15.08, *P* = 0.035), operative time > 40 min (multivariable OR: 4.15, 95% CI: 2.24–7.69, *P* < 0.001). Conversely total intravenous anesthesia was associated with lower incidence of PONV (multivariable OR: 0.40, 95% CI: 0.22–0.74, *P* = 0.003). Other factors, such as age, BMI, smoking status, ASA classification, hernia size, surgery type, operative time, and anesthesia type, were not significantly associated with postoperative pain after adjusting for other variables.

**Conclusion:**

Postoperative nausea and vomiting (PONV) are significantly reduced with total intravenous anesthesia (TIVA) compared to inhalation anesthesia, with no notable difference in postoperative pain between the two methods.

**Supplementary Information:**

The online version contains supplementary material available at 10.1007/s10029-025-03256-4.

## Introduction

Primary ventral hernias (PVH), including umbilical and epigastric hernias, are common conditions often requiring surgical intervention. Approximately 263,000 hernia surgeries performed annually in the USA alone with a significant portion of the population diagnosed annually [1, 2]. Surgical repair, particularly in cases of PVH can be performed using various techniques, with mesh repair becoming the preferred method for hernias larger than 2 cm to reduce recurrence rates [3]. However, the choice of surgical repair, including whether to use mesh, depends on factors such as hernia size and patient condition.

Postoperative pain, nausea and vomiting are common concerns following hernia repair with studies showing incidences of 20–30% [4], with both surgical and anesthetic factors contributing to patient outcomes [5, 6].

Literature reports postoperative pain in approximately 30–50% of patients following umbilical and epigastric hernia (PVH) repair, influenced by factors such as surgical technique, patient characteristics, and pain management strategies [7–10].

Ventral hernia repair is usually carried out under general anesthesia. General anesthesia is delivered either by total intravenous anesthesia (TIVA) or inhalation anesthesia [11, 12]. Inhalation anesthesia employs volatile anesthetic agents. Both strategies have benefits and drawbacks. TIVA has been shown to reduce the likelihood of postoperative nausea, while inhalation anesthesia may increase nausea but is still widely used. The choice of anesthetic technique can significantly impact patient recovery, especially concerning pain control and nausea/vomiting [12–14].

The aim of this study was to explore the impact of TIVA, inhalation anesthesia, and surgical approaches on postoperative pain and nausea following ventral hernia repair surgery. Open ventral hernia repair, being a standardized and in most cases uncomplicated procedure, may serve as a model for exploring factors influencing the risk of postoperative nausea and vomiting (PONV) and pain, with relatively few other factors influencing the outcome. The aim was thus to explore the impact of anesthesia on PONV and pain.

## Materials and methods

The study cohort was derived from the Swedish Ventral Hernia Register (SVHR), a national registry established in 2012 to collect detailed data on ventral hernia repairs conducted across Sweden. This register includes patient demographics, hernia characteristics, and surgical techniques. For the current study, we included hernia repairs performed between January 1, 2016, and December 31, 2021. Data extracted from the SVHR included patient BMI, hernia size, repair methods (e.g., mesh or sutures), and whether the surgery was elective or acute.

To further investigate anesthesia type and postoperative outcomes, the cohort was cross-referenced using Swedish National Registration Numbers with the Swedish National Register for Perioperative Care (SPOR) [15–17]. SPOR is a national quality register that records perioperative details, including anesthesia type (e.g., TIVA), intraoperative management, and postoperative outcomes such as pain, nausea, and vomiting. Through this linkage, we obtained data on postoperative pain, nausea, and vomiting for the included patients.

### Assessment of postoperative pain and nausea

Data on anesthesia type (i.e., TIVA) was retrieved from SPOR. Postoperative pain and nausea were specifically assessed and documented in the SVHR and SPOR registers. Perceived pain was recorded one hour postoperatively and at discharge. Postoperative pain was considered present if rated higher than 0 on any occasion. Nausea and vomiting were similarly recorded in the postoperative period, based on patient-reported experiences documented in the registers. Nausea and/or vomiting registered prior to discharge from the postoperative unit was considered affirmative of PONV.

### Inclusion criteria

For the study, the following inclusion criteria were applied:

- Type of Surgery: Only patients undergoing open surgical repair of ventral hernias were included, focusing on outcomes specific to open repair techniques.
- Surgical Dates: Procedures performed between January 1, 2016, and December 31, 2021, were selected to ensure consistency in data collection and reflect contemporary surgical practices.
- Availability of Complete Data: Patients with complete data on key variables, including BMI, hernia size, repair method, anesthesia type, and postoperative outcomes (pain and nausea) from both the SVHR and SPOR registers.

### Age cut-off and differentiation of patient groups

The median age of the included patient, 52 years, was used as cut-off. Similarly, a BMI cut-off of 27 kg/m² was used to identify patients at increased risk for postoperative complications. Hernias larger than 2 cm were classified as clinically significant, as they tend to exhibit different recurrence rates and complication profiles. An operative time cut-off of 40 min was chosen, as procedures lasting longer may indicate greater complexity and are potentially associated with increased postoperative risks.

### Ethical considerations

This register study was conducted in accordance with the Declaration of Helsinki and approved by the ethics review boards at the participating centres in Sweden (Diary number 2022-03564-01).

### Statistical analysis

Univariable and multivariable logistic regression analyses were conducted to assess postoperative pain as the outcome. Postoperative pain was defined as any level of pain reported either one hour after surgery or at the time of discharge. Similarly, PONV was analyzed using both univariable and multivariable logistic regression models. All statistical analyses were performed using the Statistical Package for Social Sciences (SPSS) for Windows, version 29.0, developed by IBM Corp., Armonk, NY, USA.

## Results

**Fig. 1:**
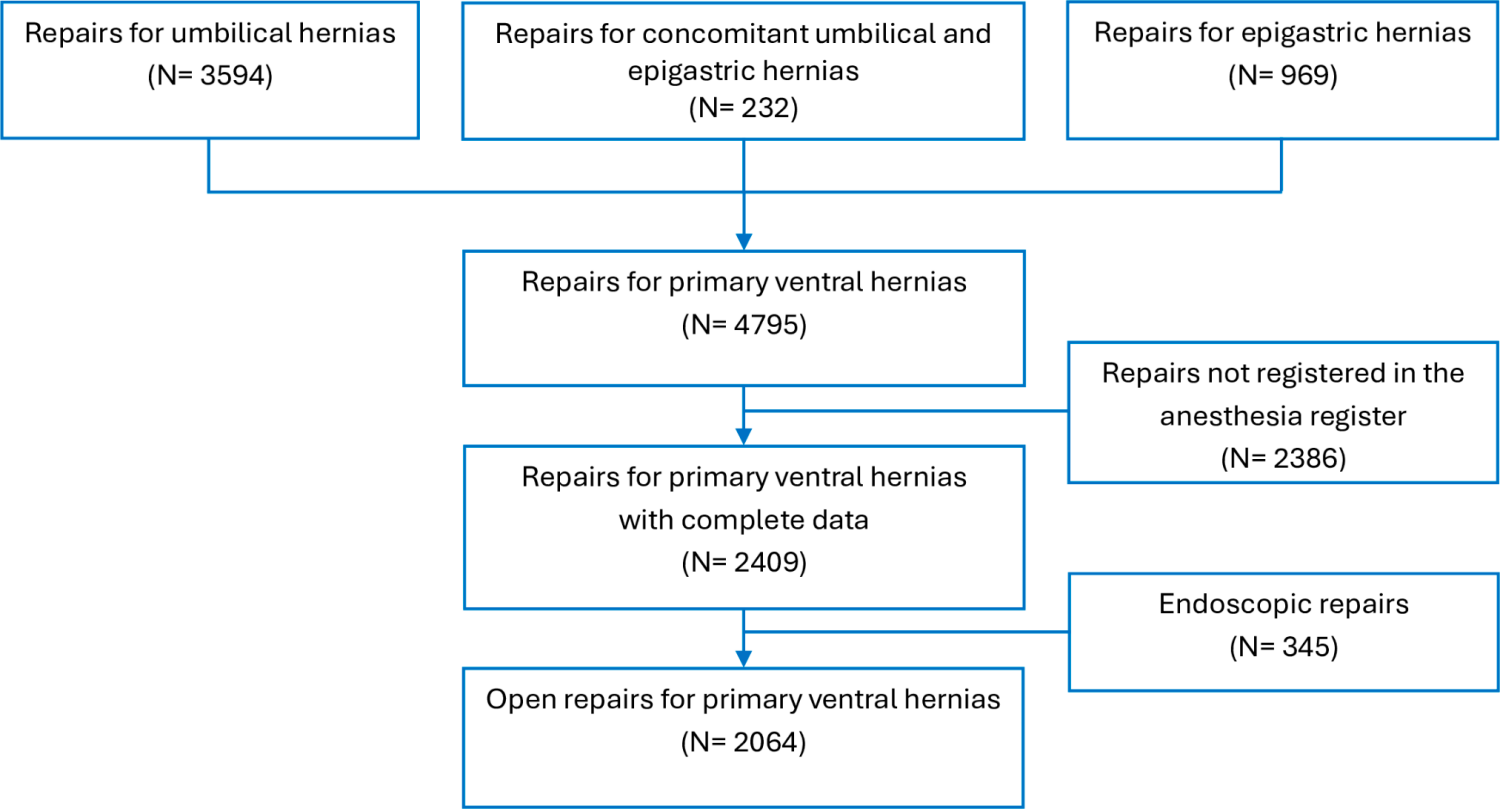
Flow chart

A total of 2,064 patients were included in the study, of which 1,248 (60.5%) were male. The distribution of hernia types was as follows: 1,069 patients (85.7%) had umbilical hernias, 150 (12.0%) had epigastric hernias, and 29 (2.3%) had combined umbilical and epigastric hernias. Among the women, 556 (68.1%) had an umbilical hernia, 217 had (26.6%) had an epigastric hernia, and 43 (5.3%) had a combined hernia, as presented in Fig. 1.

Among those who underwent elective surgery, 1,396 (78.5%) had an umbilical hernia, 316 (17.8%) had epigastric hernia, and 66 (3.7%) had a combined hernia; whereas 209 (81.3%) had emergency surgery for umbilical hernia, 43 (16.7%) had epigastric hernia, and 5 (1.9%) had a combined hernia.

Patients who underwent surgery under TIVA had umbilical hernias 559 (77.5%), epigastric hernias 139 (19.3%) and combined hernias 23 (3.2%), while those who underwent surgery under inhalation anesthesia had umbilical hernia 426 (75.9%), epigastric hernia 113 (20.1%), and combined hernia 22 (3.9%), as presented in Table 1.

**Table 1:** Baseline characteristics of patients undergoing different types of primary ventral hernia repair. ASA classification: American Society of Anesthesiologists classification

Characteristic	Umbilical hernia repair	Epigastric hernia repair	Combined hernia Umbilical, epigastric	All primary ventral hernia repair
**Gender**				
Men (%)	1069 (85.7%)	150 (12.0%)	29 (2.3%)	1248 (60.5%)
Women (%)	556 (68.1%)	217 (26.6%)	43 (5.3%)	816 (39.5%)
**Age**				
Mean, age years, (SD)	53.2 (14.9)	48.4 (15.9)	49.2 (15.2)	52.2 (15.2)
**BMI**				
Mean BMI (SD)	28.2 (4.9)	26.4 (5.5)	27.8 (7.5)	27.8 (5.2)
**Hernia size**				
Median hernia Width (cm), interquartile range	1 (1–2)	1 (1–2)	1 (1–2)	1 (1–2)
**ASA classification**				
I (%)	645 (74.6%)	188 (21.7%)	32 (3.7%)	865 (41.9%)
II (%)	729 (81.2%)	142 (15.8%)	27 (3.0%)	898 (43.5%)
III (%)	219 (83.9%)	31 (11.9%)	11 (4.2%)	261(12.6%)
IV (%)	15 (78.9%)	2 (10.5%)	2 (10.5%)	19 (0.9%)
Data missing (%)	17 (80.9%)	4 (19.0%)	0 (0%)	21 (1.0%)
**Surgery type**				
Elective surgery (%)	1396 (78.5%)	316 (17.8%)	66 (3.7%)	1778 (86.1%)
Emergency surgery (%)	209 (81.3%)	43 (16.7%)	5 (1.9%)	257 (12.5%)
Data on missing (%)	20 (68.9%)	8 (27.6%)	1 (3.4%)	29 (1.4%)
**Anesthesia type**				
Local anesthesia (%)	169 (80.4%)	32 (15.2%)	9 (4.3%)	210 (10.2%)
Spinal anesthesia (%)	6 (42.9%)	4 (28.6%)	4 (28.6%)	14 (0.7%)
General, inhalation anesthesia (%)	426 (75.9%)	113 (20.1%)	22 (3.9%)	561 (27.2%)
General, intravenous anesthesia (%)	559 (77.5%)	139 (19.3%)	23 (3.2%)	721 (34.9%)
Data missing (%)	465 (83.3%)	79 (14.2%)	14 (2.5%)	558 (27.0%)
**Operative time**				
Mean operative time, minutes (SD)	44 (43)	53 (39)	81 (48)	52 (41)

The study showed that PONV were reported by 27.5% of patients. In terms of postoperative outcomes in multivariable and univariable regression, gender, age, operative time, and type of anesthesia significantly influenced the incidence PONV. Men were significantly less likely to experience nausea or vomiting compared to women, with odds ratios of 0.37 (*p* < 0.001) in the univariable analysis and 0.30 (*p* < 0.001) in the multivariable analysis. Patients older than 52 years had an odds ratio of 0.54 in the univariable analysis, but this was not significant in the multivariable analysis (odds ratio of 0.50, *p* = 0.277).

Overall, gender, type of surgery, operative time, and type of anesthesia significantly influenced outcomes, while age, body mass index (BMI), smoking, ASA classification, and hernia size had limited effects as shown in Table 2.

**Table 2:** Univariable and multivariable logistic regression analysis with postoperative nausea as outcome. CI: Confidence Interval. ASA classification: American Society of Anesthesiologists classification, TIVA: Total Intravenous Anesthesia. reference: the comparison group used for calculating odds ratios

Variable	Univariable Odds ratio (95% CI)	*P*-value	Multivariable Odds ratio (95% CI)	*P*- value
**Gender**				
Women (Reference)	Reference		Reference	
Men	0.37 (0.24–0.58)	< 0.001	0.30 (0.18–0.49)	< 0.001
**Age**				
<52 years old (Reference)	Reference		Reference	
>52 years old	0.54 (0.35–0.83)	0.005	0.50 (0.15–1.74)	0.277
**BMI**				
BMI < 27 (Reference)	Reference		Reference	
BMI > 27	1.11 (0.72–1.72)	0.639	1.25 (0.39–3.94)	0.711
Smoker, OR	0.50 (0.16–1.61)	0.248	01.31 (0.35–4.89)	0.691
**ASA classification**				
I (Reference)	Reference		Reference	
II	0.84 (0.53–1.34)	0.469	0.60 (0.17–2.15)	0.434
III	1.01 (0.54–1.91)	0.969	1.65 (0.34–7.93)	0.535
IV				
**Hernia size**				
Hernia < 2 cm (Reference)	Reference		Reference	
Hernia > 2 cm	1.63 (0.38–7.07)	0.514	0.81 (0.17–4.2)	0.799
**Surgery type**				
Elective surgery (Reference)	Reference		Reference	
Emergency surgery	2.84 (1.76–4.6)	< 0.001	4.08 (1.10-15.08)	0.035
**Operative time**				
<40 min (Reference)	Reference		Reference	
> 40 min	3.20 (1.98–5.23)	< 0.001	4.15 (2.24–7.69)	< 0.001
**Anesthesia type**				
Inhalation A. (Reference)	Reference		Reference	
TIVA	0.30 (0.17–0.53)	< 0.001	0.40 (0.22–0.74)	0.003

Postoperative pain was reported by 34.6% of patients. Multivariable regression analysis showed that gender was the only significant factor, with females experiencing more postoperative pain than males. In univariable regression, both age and gender were found to significantly influence pain levels as shown in Table 3.

**Table 3:** Univariable and multivariable logistic regression analysis with postoperative pain as outcome. CI: Confidence Interval. ASA classification: American Society of Anesthesiologists classification, TIVA: Total Intravenous Anesthesia. reference: the comparison group used for calculating odds ratios

Variable	Univariable Odds ratio (95% CI)	*P*- value	Multivariable Odds ratio (95% CI)	*P*-value
**Gender**				
Women (Reference)	Reference		Reference	
Men	0.65 (0.45–0.94)	0.021	0.60 (0.44–0.83)	0.002
**Age**				
< 52 years old (Reference)	Reference		Reference	
> 52 years old	0.59 (0.41–0.87)	0.007	0.68 (0.30–1.57)	0.369
**BMI**				
BMI < 27 (Reference)	Reference		Reference	
BMI > 27	1.15 (0.78–1.68)	0.482	1.19 (0.52–2.74)	0.683
Smoking (Yes/No)	1.40 (0.71–2.73)	0.336	1.31(0.35–4.89)	0.691
**ASA classification**				
I (Reference)	Reference		Reference	
II	1.21 (0.79–1.83)	0.379	1.86 (0.73–4.73)	0.195
III	1.36 (078-2.38)	0.285	2.45 (0.63–9.54)	0.196
IV				
**Hernia size**				
<2 cm (Reference)	Reference		Reference	
>2 cm	0.75 (0.31–1.86)	0.541	0.72 (0.26–2.01)	0.532
**Surgery type**				
Elective surgery (Reference)	Reference		Reference	
Emergency suegery	0.83 (0.46–1.50)	0.542	1.00 (0.30–3.34)	0.997
**Operative time**				
< 40 min (Reference)	Reference		Reference	
> 40 min	0.96 (0.66–1.38)	0.822	0.81 (0.36–1.80)	0.599
**Anesthesia type**				
Inhalation A. (Reference)	Reference		Reference	
TIVA	0.83 (0.55–1.25)	0.374	0.78 (0.33–1.84)	0.562

## Discussion

### Postoperative nausea and vomiting

PONV are common and adverse effects following surgery and anesthesia [18], after surgery for primary ventral hernias as well as after more complex procedures. The risk factors for developing PONV can be grouped into three categories: patient-related, anesthetic-related, and surgical-related factors.

In the present study, patient-related factors contributing to PONV were identified as female gender, and age below 52 years was identified as a patient-related factor possibly contributing to PONV, but that this factor did not remain significant after adjusting for other variables in the multivariable analysis. Anesthetic-related factors included the use of inhalation anesthesia and emergency surgeries. Surgery-related factors were longer procedures and emergency surgeries. The focus of this study was to explore a proactive approach to managing postoperative pain and nausea following surgery for primary ventral hernias.

Despite the increased use of preventive anti-nausea drugs and multi-modal pain relief treatments, PONV still affects approximately 30% of patients undergoing elective surgery, with some high-risk patients experiencing rates as high as 70% [18, 19].

The results in the current study showed a lower risk of postoperative PONV in patients undergoing hernia repair with TIVA compared to those undergoing hernia repair with inhalation anesthesia. Gender, surgery type, and surgery duration were the most important factors. Men were less likely to experience nausea compared to women, with a much lower odds of nausea (OR: 0.30, 95% CI: 0.18–0.49, *p* < 0.001). While aged below 52 years appeared to contribute to PONV risk in unadjusted analysis, it was not a significant factor in the multivariable model. BMI and smoking did not show a significant impact on nausea risk in either analysis. However, patients who had emergency surgeries and those whose surgeries lasted longer than 40 min were at a higher risk.

### Postoperative pain

In the present study we analyzed factors related to postoperative pain using both univariable and multivariable analyses. The results indicated that gender and age were crucial factors. Men were less likely to experience postoperative pain compared to women, with an odds ratio of 0.60 (95% CI: 0.44–0.83, *p* = 0.002). Although patients over 52 years old showed a lower risk of pain in the univariable analysis (OR: 0.59, 95% CI: 0.41–0.87, *p* = 0.007), this association was not significant after adjusting for other factors (*p* = 0.369).

Factors like BMI, smoking, ASA classification, hernia size, surgery type, operative time, and anesthesia type did not have significant effect on postoperative pain. For example, patients with a BMI over 27 and those who smoked had odds ratios close to 1, indicating no real connection to pain risk. Similarly, emergency surgeries and the duration of surgery did not impact pain levels.

Risk factors such as high BMI, low age, and female gender should be taken into consideration for optimal management of postoperative pain [10, 12]. However, this study did not show a significant effect on postoperative pain. It is possible that enhanced recovery pathways, preoperative counseling, and patient education could mitigate postoperative pain and nausea, but further studies are needed to confirm this.

The present study was conducted as an observational study, which may have been a source of bias. There may have been residual confounding, especially related to the choice of anesthesia. The ventral hernia register includes detailed data on the hernia anatomy and patient-related factors, but the method of anesthesia may have been chosen based on preoperatively predicted risks of pain and PONV related to factors not registered in the perioperative register. The choice of anesthesia is also a matter of local traditions, which may have affected the outcome.

### Strengths and limitations

#### Strengths

This study is based on national registry data, which boasts a high degree of coverage. The comprehensive nature of the data allows for robust insights into the specific population and surgical practices within our region. This high coverage rate provides a solid foundation for the study’s findings and enhances their internal validity.

#### Limitations

This study has several limitations that should be considered when interpreting the results. Firstly, the reliance on data collected during routine clinical practice may have led to the exclusion of pertinent variables not recorded in the registry, which could influence postoperative outcomes. Significant changes in surgical practices and anesthesia techniques may have occurred during the study period, potentially affecting variability in the results. This temporal bias could affect the consistency of outcomes and complicate comparisons across the analyzed time frame. While the study accurately reflects a specific population from our healthcare region, generalizability to other healthcare systems or settings may be limited. Variations in patient demographics, surgical protocols, and anesthesia practices across regions can influence outcomes, so caution should be exercised when applying these findings to different patient groups or healthcare contexts.

## Conclusion

PONV were less common in men, shorter surgeries, and with TIVA, while emergency surgeries and longer operative times increased PONV risk. Postoperative pain was more frequently reported by women. Other factors like age, BMI, and smoking did not significantly affect PONV or pain outcomes. Gender, surgery type, operative time, and anesthesia were the main predictors of postoperative issues.

## Electronic supplementary material

Below is the link to the electronic supplementary material.

Supplementary Material 1

## Data Availability

Not applicable.
